# Clinician and patient experiences when providing and receiving information and support for managing chemotherapy‐induced peripheral neuropathy: A qualitative multiple methods study

**DOI:** 10.1111/ecc.13517

**Published:** 2021-10-12

**Authors:** Mary Anne Lagmay Tanay, Glenn Robert, Anne Marie Rafferty, Rona Moss‐Morris, Jo Armes

**Affiliations:** ^1^ Florence Nightingale Faculty of Nursing, Midwifery and Palliative Care King's College London London UK; ^2^ Institute of Psychiatry, Psychology and Neuroscience King's College London London UK; ^3^ School of Health Sciences, Faculty of Health and Medical Sciences University of Surrey Guildford UK

**Keywords:** cancer, chemotherapy, chemotherapy‐induced peripheral neuropathy, CIPN, qualitative

## Abstract

**Objective:**

To improve patient experience of chemotherapy‐induced peripheral neuropathy (CIPN), it is crucial to identify how patients develop their understanding and perception of CIPN. A wider understanding of the experiences of clinicians who provide CIPN information and support is also needed. This study explored clinician and patient experience of the provision of care, information and support for CIPN.

**Methods:**

Data were collected between July and November 2019 using multiple qualitative methods. Non‐participant observations were undertaken in colorectal and breast cancer clinics and at clinician stations, including the observation of chemotherapy consultations between patients and clinicians. Semi‐structured interviews with people with cancer and clinicians were also conducted. Data were analysed using inductive reflexive thematic analysis.

**Results:**

Three major themes emerged: (1) CIPN is a hidden chemotherapy side effect, (2) assessment and management of CIPN is disconnected and (3) patients and clinicians expect openness in CIPN symptom reporting, information provision and management.

**Conclusion:**

Findings show the need to address the lack of patients' overall familiarity with CIPN. Echoing earlier studies, our findings suggest that knowledge and understanding about CIPN among clinicians are limited or lacking. These insights from patient and clinicians' CIPN experiences can inform future interventions that may address the genuine needs of patients and enhance CIPN support.

## 1 BACKGROUND

Chemotherapy‐induced peripheral neuropathy (CIPN) has a negative effect on a person's quality of life (Beijers et al., 2014; Gordon et al., 2018; Tanay et al., 2017). Subjective, invisible symptoms such as numbness, tingling and pain in the hands, feet or both are the most frequently reported CIPN symptoms (Gordon‐Williams & Farquhar‐Smith, 2020; Park et al., 2013; Staff et al., 2017). CIPN symptoms affect physical function and can reduce ability to perform social, domestic, and work activities (Tanay et al., 2017). Such physical impairment can result in emotional and psychological issues such as anxiety, low mood and inability to cope (Tofthagen, 2010). The dominant management approach is to delay, reduce or discontinue treatment to allow CIPN symptoms to improve. Early patient reporting of CIPN symptoms, assessment by clinicians and early management are key to preventing severe CIPN symptoms or permanent nerve damage (Jordan et al., 2020; Knoerl et al., 2018; Loprinzi et al., 2020). However, people who experience CIPN are unable to describe their symptoms clearly and frequently use metaphors (Tanay et al., 2017), which may hamper symptom assessment by clinicians.

There are few studies exploring clinician experiences of providing information and support for managing CIPN. An American study which analysed audio‐recordings of outpatient clinic consultations showed that clinicians discussed and documented CIPN in less than half of their clinical encounters with patients at risk of developing CIPN (Knoerl et al., 2019). Studies indicate that nurses reported they lacked CIPN‐specific knowledge (Al‐Atiyyat & Banifawaz, 2018; Binner et al., 2011) alongside limited understanding of the neurotoxic nature of particular chemotherapies and evidence‐based CIPN management (Smith et al., 2014). In one UK survey with multi‐disciplinary clinicians, participants reported they lacked knowledge of CIPN local services, and most reported dissatisfaction with current local CIPN management (Taylor & Tanay, 2020).

It is important to identify how patients develop their understanding and perception of CIPN. The information discussed during patient–clinician interactions concerning CIPN, factors that influence these interactions and the accessibility of CIPN services can all influence patients' overall experience. To improve patient experience, a wider understanding of clinician experiences in providing CIPN information and support is also needed. To date, no study has explored clinicians' and patients' perspectives of their shared experience of CIPN. Consequently, little is known about how patient and clinician perspectives, separately or in combination, influence both patient and clinician behaviours concerning the provision and reception of CIPN information and support. Using multiple qualitative methods, this study aims to explore the experiences of patients and clinicians in relation to the provision of care, information and support for CIPN.

## 2 METHODS

### 2.1 Study design

This study is part of a larger Experience‐Based Co‐Design (EBCD) study using an approach that draws upon the concepts of ‘user involvement’ and ‘user experience’ for service improvement or intervention development (Bate & Robert, 2007). A qualitative research methodology, combining observation and interview data collection methods, was employed to explore individuals' experiences of information, provision of care and support for CIPN. Observations allowed the researcher to witness what participants did, what they said and how patients and clinicians interacted or behaved (Green & Thorogood, 2018) during pre‐chemotherapy consultations and subsequent chemotherapy consultations when CIPN information, assessment and management were discussed. Semi‐structured interviews allowed participants to share accounts or perceptions of their experiences (Cresswell, 2014; Green & Thorogood, 2018).

#### 2.1.1 Participants

Following ethical approval and authorisation from a local research and development office, patient participants were recruited from outpatient oncology clinics in an NHS hospital in London. Purposive convenience sampling was used to identify potential patient participants booked in certain clinic days who were initially approached by their clinical team. Inclusion criteria were as follows: ≥18 years old, colorectal or breast cancer diagnosis and about to have treatment or has been treated with neurotoxic chemotherapy. Patients who had pre‐existing neuropathy due to other causes, such as diabetes, were excluded. If they agreed, they were introduced face‐to‐face to the lead researcher (M. T.) who provided information about the study. Clinicians were recruited from the oncology directorate. They were invited to participate if they were a permanent member of staff who was involved with information‐giving about chemotherapy, including assessment and management of CIPN. All participants gave written consent to participate and were given the option to be interviewed, observed or both. Due to busy workload and time constraints, chemotherapy unit clinician participants decided to undergo group interview instead of being interviewed individually.

#### 2.1.2 Data collection

Data collection was conducted between July to November 2019 by M. T., a female oncology nurse researcher with previous experience and training in qualitative research methods. Thirteen episodes of non‐participant observations were undertaken (Green & Thorogood, 2018)—the researcher did not actively take part in the interaction—over a total of 39 h in the colorectal (Thursdays 1:30 PM–4:00 PM) and breast cancer clinics (Wednesdays 9:30 AM–12:00 PM). Thirty‐four hours was conducted in the outpatient unit clinician station (staff hub), and 5 h was conducted in clinic rooms during chemotherapy consultations between patients and clinicians. Consultations were audio‐recorded and transcribed if both patient and clinician consented. M. T. documented field notes. Table 1 shows examples of the observation schedule and field notes. If a patient was accompanied by a friend or relative, they provided verbal consent before observation of the consultation began.

**TABLE 1:** Examples of observation schedule, field notes and qualitative interview questions

Observation schedule	Field note example
Consultation general information Date and time Clinician and patient study identifier Stage of chemotherapy (before treatment, ongoing treatment, end of treatment) Chemotherapy drug and cycle number Time in and time out Pre‐chemotherapy consultation Setting description Preparation of clinician specific to CIPN before seeing patient e.g. pre‐clinic discussion with the clinical team, forms of reminder Nurse verbal and non‐verbal communication Patient verbal and non‐verbal communication How patients described and reported their symptoms Who initiated CIPN discussion Percentage of time when CIPN was discussed by clinicians How CIPN was assessed or discussed by clinicians in the context of other chemotherapy side effects Written CIPN resources given to the patient Content and nature of CIPN discussion Clinician actions, referrals or prescriptions (specific to CIPN) made after the consultation General notes Date and time when field notes were transcribed Researcher's reflections	19 September 2019 (Outpatient clinics) 1345‐1410 I attended the pre‐clinic clinician meeting held in one of the meeting rooms in the outpatient clinic. It was attended by three medical oncologists and two clinical nurse specialists. Patient summaries that clinicians go through in pre‐clinic meetings have notes such as: Dose reduction, history of PN History of neuropathy from previous cycles Grade 3 peripheral neuropathy affecting mobility. Has been gradually getting worse since FOLFOX. Plan: Proceed Cycle 2 with dose reduction oxaliplatin (this patient is for Cycle 3 next week) Hold oxaliplatin from this cycle Clinical review 4/52 and if ongoing neuropathy, to hold future oxaliplatin from rest. Reduce oxaliplatin Monitor neuropathy Some discussions such as ‘If no neuropathy, then proceed to cycle 12. If with PN, tell patient it is okay to stop.’ 1420 I was invited by a doctor to see a patient to give a participant information sheet and tell the patient about my study. This was after she was seen by the doctor who gave information about chemotherapy. As we were going in, I was introduced by the doctor. She was then given two PIS which contain information about chemotherapy which were printed from a cancer charity website (CAPOX and FOLFOX). As we came in, the doctor mentioned to the patient about ‘pins and needles’, nerve damage by oxaliplatin. ‘Nerves take long time to recover. So this can be longer to get better or may not improve at all. If you have this, we may stop or reduce your dose’ 1426 The doctor left me in the room with the patient, I gave the study PIS. The clinical nurse specialist then saw her with the pack. Reflection: The patient was already given chemotherapy information but the doctor gave additional information about CIPN when we came in the clinic room together. Perhaps my presence reminded him of CIPN. (Field notes were transcribed immediately after clinic.)
Examples of semi‐structured interview questions	
Clinicians	
Are there any factors that influence the main priorities/topics during chemotherapy consultation? What are these? What key messages would you like the patient to remember after chemotherapy consultation? Can you please describe how you give information about peripheral neuropathy to patients? Please describe how you feel about CIPN. What will help when giving information to patients about CIPN?	
Patients	
How did you find the pre‐chemotherapy consultation? What were the key take‐home messages for you? What side effects of chemotherapy stood out for you, if any? Why do you think this/these stood out for you? Can you please tell me your understanding of peripheral neuropathy as a possible side effect of your treatment? What were the key take home messages for you about this particular side effect? How do you feel about this side effect?	

A topic guide with open‐ended and probing questions and pilot tested with patient representatives (shown in Table 1) was used for the semi‐structured qualitative interviews, which lasted between 19 and 45 min. All audio‐recorded patient and clinician interviews were conducted by M. T. in a single clinic room; recordings were transcribed verbatim.

#### 2.1.3 Data analysis

The inductive reflective thematic analysis (RTA) process described by Braun and Clarke (Braun et al., 2019) was used for the analysis. RTA allows the identification of meaning‐based patterns through a rigorous process of data familiarisation; data coding using MS Excel; theme development and revision; and theme refinement, definition and naming. It acknowledges the active engagement of the researchers in the data interpretation and knowledge production (Braun et al., 2019). M. T. coded the interview and observational data. G. R., J. A. and A. M. R. were involved in generating the themes. All researchers discussed their description and interpretation of the emerging themes. This iterative process of revision and refinement continued until consensus among the researchers was reached. The process was completed separately for the patient interviews, clinician interviews and observational data. To form the final themes, the themes from each data source were compared to determine similarities, differences and relationships. Triangulation of different data sources during analysis enabled validation of identified themes (Nowell et al., 2017).

## 3 FINDINGS

In total, 15 clinicians and 12 patients consented to participate. Participant characteristics and details of their participation in consultation observations, interviews or both are shown in Table 2. Nine clinician and 11 patient semi‐structured interviews (Cresswell, 2014) were conducted, and a group interview with four nurses was also conducted. Nine patient–clinician chemotherapy consultations were observed.

**TABLE 2:** Characteristics of the study participants

Patient participants								
Study identifier	Gender	Age band	Ethnicity	Cancer diagnosis	Chemotherapy intent	Neurotoxic drug	Chemotherapy treatment stage during data collection	Interviewed (I) or observed (O)
P‐01	Male	70	White	colon	Adjuvant	Oxaliplatin	Before treatment	I, O
P‐02	Female	40	White	Breast	Adjuvant	Paclitaxel	Midway	O
P‐03	Female	50	Black	Breast	Adjuvant	Paclitaxel	Before treatment	I, O
P‐04	Female	70	White	Breast	Adjuvant	Paclitaxel	Before treatment	I, O
P‐05	Female	50	White	caecum	Palliative	Oxaliplatin	End of treatment	I, O
P‐07	Female	60	Black	Breast	Adjuvant	Paclitaxel	End of treatment	I
P‐08	Female	60	White	Breast	Adjuvant	Paclitaxel	End of treatment	I, O
P‐09	Female	60	White	colon	Adjuvant	Oxaliplatin	Midway	I, O
P‐10	Female	70	Black	colon	Adjuvant	Oxaliplatin	End of treatment	I
P‐11	Female	30	White	Appendix	Adjuvant	Oxaliplatin	End of treatment	I, O
P‐12	Female	30	Mixed White‐Asian	colon	Adjuvant	Oxaliplatin	End of treatment	I, O
P‐13	Female	60	White	colon	Palliative	Oxaliplatin	End of treatment	I

Clinicians				
Study identifier	Gender	Job role	Interviewed (I), observed (O), interviewed in a group (IG)	
C‐01	Female	Cancer nurse specialist	I, O	
C‐02	Female	Cancer nurse specialist	O	
C‐03	Female	Senior chemotherapy nurse	O	
C‐04	Female	Cancer specialist pharmacist	I	
C‐05	Male	Cancer specialist pharmacist	I, O	
C‐06	Female	Cancer nurse practitioner	I, O	
C‐07	Female	Occupational therapist	I	
C‐08	Female	Senior chemotherapy nurse	I, O	
C‐09	Male	Senior medical oncologist	I, O	
C‐10	Female	Senior chemotherapy nurse	IG	
C‐11	Female	Senior chemotherapy nurse	IG	
C‐12	Female	Junior chemotherapy nurse	IG	
C‐13	Female	Senior chemotherapy nurse	IG	
C‐14	Male	Junior medical oncologist	I, O	
C‐15	Female	Physiotherapist	I	

**FIGURE 1:**
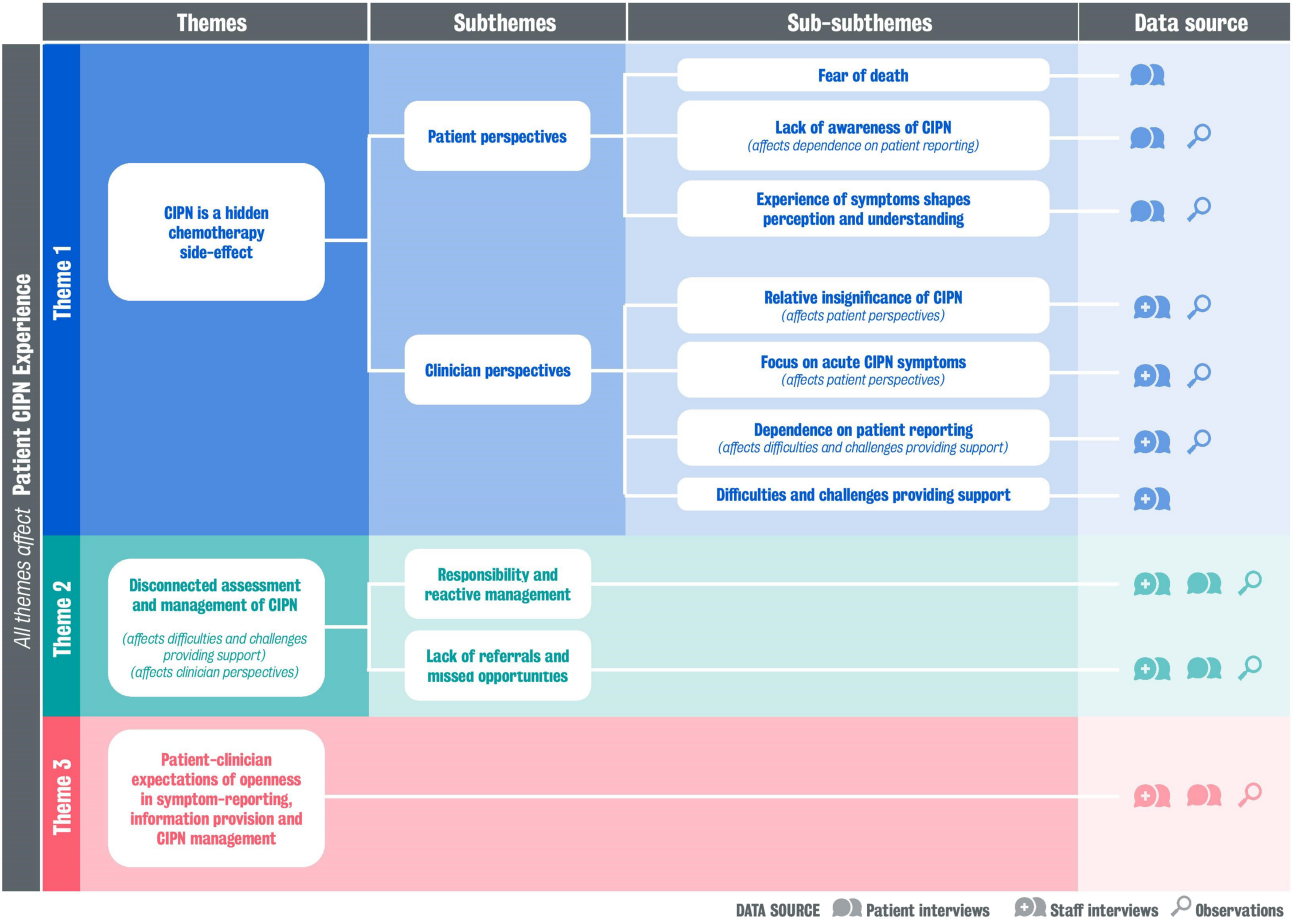
Representation of experiences of clinicians and patients of the provision of care, information and support for chemotherapy‐induced peripheral neuropathy

Three major themes emerged: (1) CIPN is a hidden chemotherapy side effect, (2) assessment and management of CIPN is disconnected and (3) patients and clinicians expect openness in CIPN symptom reporting, information provision and management. Illustrative participant interview quotes are presented in Tables 3 and 4. Data extracts are presented using participant identifiers P (patient), C (clinician) and FN (field notes). The themes and subthemes as well as the relationship of these to the overall patient experience are represented in Figure 1.

**TABLE 3:** Representative participant interview quotes (Theme 1)

Themes	Subthemes	Participant quotes
Theme 1: CIPN is a hidden chemotherapy side effect	Patient perspectives	
	Fear of death	‘I was very eager to get into chemo because I know I need it so badly. I was ready to face all the side effects just because I knew I needed it. Looking at the list you know, it was a very substantial list. So obviously I was a bit like “Okay, this could be really difficult”, but I was just ready to do whatever the side effects were.’ (P‐11) ‘In the beginning, you are so frightened and you have just had this massive, big blow that's blown your whole world apart, saying what you have got and you have more fear of death than fear of medication… I just thought they did not give me the last one, I would not get neuropathy, but I was thinking if they do not give me the last one, then my cancer's going to grow quick. I was more frightened of not having it (chemotherapy) than having it.’ (P‐05)
	Lack of awareness of CIPN	‘I had no idea that neuropathy even existed before knowing that I needed to have chemotherapy and that that was one of the side effects of it. All of them, my friends and family were very shocked when I told them that it was a thing that could happen as a result of the chemotherapy.’ (P‐12) ‘It was a new concept. I had not heard of that as a side effect with cancer treatment. But I guess in the list of side effects I usually discuss with the doctor, it is quite near I guess the bottom because it's like it's not an obvious one.’ (P‐11) ‘I did not understand that it was going to be like it was. When I left it was a colder day and it was raining and when the hands get wet and you push a door or something like that and then shock (moves hands) “What's going on?”.’ (P‐01) ‘Do I report them as well? I thought it (CIPN) was normal’ (P‐03)
	Experience of symptoms shapes perception and understanding	‘The word numb does not do it. Do you know what I mean? You have to be in my toes to know what it feels like.’ (P‐08) ‘Not really, I did not exactly know, but obviously I do now because I'm going through it. Obviously my feet… it's a really odd feeling. I cannot even explain it. I'd probably try and explain it the best I can. I cannot even explain it to you because me explaining it to you, you'd have to experience what I'm feeling. But it's just a weird feeling, it's just not nice. No, it's not a nice feeling at all. I am aware now that it's there. I try to forget about it or put it at the back of my mind. Even I'm talking to you, it's there. There's nothing I can do.’ (P‐07) ‘They're just moving around like insects. They're now on the feet. They gave lots of reading material but I think the experience and reading are two different things.’ (P‐10) ‘And I just sort of did not think of it as anything to report. But now because it's happened a few times, I feel like it is chemo related. I guess it's because it's not one of the high profile things that you think about when you think about chemotherapy, like the nausea, the vomiting, the hair loss. It's not so like well‐known. But I think it was on the list of possible side effects I got in the handout, but it wasn't one that I paid particular attention to I guess.’ (P‐11) ‘I would say that if your fingers or toes feel at all numb at any point, then do not wait for the next consultation. I would say phone up and let somebody know. I think I was a bit slow. I know I was a bit slow. I do not like to be a trouble and it wasn't hurting me. It wasn't severe. I thought, “ah, it's fine. I'll just wait because it might not be anything anyway.” I think it's probably better to err on the side of assuming that it probably is. I think if I made a mistake anywhere, that was it probably (for not reporting sooner).’ (P‐08) ‘Yeah, because I've got no other ailments except neuropathy now, and it's affected me the worst of all my two years, it's worse than having cancer because it's changed my lifestyle. I know cancer changes your lifestyle, but this has done it a bit more, pushed it a bit more over the boundary…’ (P‐05)
	Clinician perspectives	
	Relative insignificance of CIPN	‘I will list the important ones like the temperature, sepsis and infection; and then use the checklist as I go through (the list of side‐effects).’ (C‐10) ‘Obviously, we worry about lots of other side effects of chemotherapy. There are more life‐threatening side effects we worry about, but I think neuropathy is one that we tend to worry about. I worry because the key thing I know is that I've seen patients that have finished treatment and they are still having neuropathy months down the line. I think what patients might not realise is how lasting the effects can be.’ (C‐14)
	Focus on acute CIPN symptoms	‘At the start, patients cannot take it all on and they are most worried about the immediate treatment and what's going to happen, not the longer term side.’ (C‐08) ‘I do not generally give timeframes (about CIPN) because, if I'm honest, that would be an area where I would not know so much but also, I think everyone is different. Some people it does take a bit longer.’ (C‐06)
	Dependence on patient reporting	‘And then you say “oh, have you mentioned to anyone?”. “Oh no, I do not want to mention it because I'm a bit worried that they'll stop my treatment” or something like that.’ (C‐07) ‘I think the things like for me I would struggle with the self‐management area. So with other side effects I could suggest things for patients at home. The difficulty is you are very much reliant on how someone has assessed a patient, reliant on the patient reporting their symptoms; and if it's bad and we do not get on top of it, the patients can be left with side effects for years.’ (C‐06) ‘The patient should be confident to recognise the side effects and let us know, for me this is the goal’ (C‐09)
	Difficulties and challenges providing support	‘I think a lot of clinicians are worried about frightening patients and I think a lot of clinicians are worried about patients refusing treatment.’ (C‐08) ‘I think it's important to tell the GP but then again, I guess the reason we do that is that I feel like we do not have anything to offer them. I do not know of anything we can offer them to help them. They get abandoned a bit, I think’ (C‐14)

**TABLE 4:** Representative participant interview quotes (Themes 2 and 3)

Themes	Subthemes	Participant quotes
Theme 2: Assessment and management of CIPN is disconnected	Responsibility and reactive management	‘Hopefully the doctors before they get to us but then they sometimes forget to tell the doctor and then they'll tell us extra things that they either forget they need to tell the doctor that or they just were not aware so they'll just tell us that and then we relay it back to the team (doctors).’ (C‐13) ‘I try and explain that actually if the symptoms are getting worse we would try and reduce doses and then we would hope to see symptoms would alleviate. I'm going to be honest, I do not really know what I would say to patients as far as self‐managing for peripheral neuropathy if I'm honest apart from informing us that if they have got worsening symptoms or having symptoms. Other things? Diarrhoea, I got that. Nausea and fatigue I could definitely do but not neuropathy.’ (C‐06) ‘I think when it gets to grade two, they understand the seriousness of what it's doing. I also explain that it's likely that it's going to get much worse if we continue treatment. It's not always their choice but I say quite seriously, “if we do not stop this, it's going to get worse.” The fact that I say that sometimes it can be permanent, I think that means that patients tend to reluctantly agree. That's my feeling.’ (C‐09)
	Lack of referrals and missed opportunities	‘Patients will tell people symptoms at different times. It's not always necessarily going to be in our consultation. Patients will tell symptoms at odd times when you will not expect it.’ (C‐14) ‘I know that's something that can be done but I've got to be honest, I do not know much about what physiotherapy can do in that situation. I think that's probably quite a big gap in my education and knowledge about what we should be doing with these patients, apart from the avoidance and reducing the dose. I do not know of any services or anything. I think it would be good to learn more about management strategies… what we do not have, or I feel like we do not have, is a set (CIPN) protocol, or a set person, or a set team that I know I can contact if I'm worried about someone’ (C‐14). ‘I know that they do something to do with pain with peripheral neuropathy. Physiotherapy, I am not 100% sure exactly. I do not know what the service is but that would be something that I would refer…’ (C‐01) ‘I think from my discussions with a lot of my colleagues from that side of things, they often just feel that it's a symptom that we know comes on and I guess because there's no clear treatment plan for it, it just sort of thought that “well, we'll just make do” kind of. So that's why I feel and from my discussions, that's why I can pick up on why we do not get as much of a referral rate for them.’ (C‐07) ‘Physiotherapy, I'm not 100% sure exactly what it is. I know that they do something to do with pain with peripheral neuropathy. I do not know what the service is but that would be something that I would refer through electronic patient record.’ (C‐06) ‘I do not think they explore the neuropathy, the nursing staff, just before the treatment, because the doctor or the prescriber who see the patients before every cycle have to deal with that.’ (C‐09)
Theme 3: Patients and clinicians expect openness	‘It's a very much an honesty the best policy syndrome. I guess you try to offer them the information about, say neuropathy when you use platinum compound or even bortezomib—It is that transient to permanent zone of experience. And making them appreciate the fact that they will not be alone. That dose reduction is about safety profile, and not because of their inability.’ (C‐05) ‘I suppose something like peripheral neuropathy is not that urgent but it's important… to report sort of urgent symptoms but also to keep a record of symptoms that we need to know about but maybe aren't urgent… I think it's about being honest about what could happen, and this is why and working with patients because I do not know if patients will withhold that information or not… I'd say it would be a joint decision (treatment modification). I have had patients who have not wanted to stop or reduce the dose and that is quite difficult. But then it is about having an adult conversation with that person and I suppose it's about being honest. So, you know I would tend to say to people, “It's no good us carrying on if you start falling over and you cannot walk properly, we do not want to leave you like that at the end of this treatment.”’ (C‐08) ‘They only told me “you might suffer”. No, I do not think they did tell me to be fair. I think I found out myself on the internet. I did ask how long, and he did not really say anything. He said “it could be a couple of months, it could be up to two years, it might be permanent”. I've read it can be permanent… there should be someone to tell you exactly what drugs you are taking, how they can affect you and a bit more. Obviously, because it's medical terms we do not understand, it's going over your head, so I think in layman's terms, it should be someone to support you more on telling you about the chemo.’ (P‐05) ‘I cannot remember whether they said its numbness or tingles, tingling in your hands and feet. I do not know, they did not—Nowhere sort of says how to sort of deal with it, do your exercise or anything, I do not know or do you just put up with it? I do not really know.’ (P‐03) ‘For me because of my understanding of how I use my hands and how I work. Making that decision [dose reduction] was quite straightforward, like I was quite determined I was resolute, made sure obviously that I had the right information, and I asked all the right questions, which is why they reduced some of my chemo’ (P‐12)	

### 3.1 Theme 1: CIPN is a hidden chemotherapy side effect

Aside from the invisible nature of CIPN symptoms, there were several patient and clinician perspectives that hindered CIPN assessment and management before, during or after chemotherapy treatment.

#### 3.1.1 Patient perspectives

##### Fear of death

Being faced with cancer which can be fatal if left untreated means CIPN is not a key patient concern at the start of treatment. Their fear of death compelled them to accept the side effects of treatment.

##### Lack of awareness of CIPN

Prior knowledge of CIPN was lacking among patient participants. Some said it was a chemotherapy side effect that they had never heard of before. Their friends, family and work colleagues were also unaware of CIPN and did not understand the symptoms. At the start of chemotherapy treatment, most patients identified hair loss as the most worrisome side effect. Only one mentioned CIPN, due to their employment which involved the use of fine hand movements. Patients who agreed to be observed during their pre‐chemotherapy consultations with a chemotherapy nurse were given verbal and written information about CIPN. However, in interviews only a few days afterwards, patients said that they did not fully understand what CIPN was and were unaware of the need to report CIPN symptoms.

##### Experience of symptoms shapes perception and understanding

It was clear that patients' understanding and perception of CIPN changed as they experienced symptoms and moved through their chemotherapy treatment. Many acknowledged that verbal and written information given to them by their clinicians ‘gives a clue’ (P‐08, P‐10) but it was only when they experienced it that they really understood CIPN symptoms (P‐07). They believed they had to experience CIPN to be able to understand it. Patients drew analogies with other experiences to describe what the symptoms felt like. They used phrases such as ‘difficult colour zones that grow and (zoom) back again’ when describing numbness (P‐08) and comparing clinician descriptions of CIPN as pins and needles to ‘being stung by sea lice’ (P‐09) and ‘there is a pattern’ when referring to CIPN frequency (P‐12). Depending on intensity, severity, and effect on daily life, CIPN symptoms attracted varying degrees of attention. Symptoms prompted patients to adopt coping strategies both at home and work. For example, one participant became aware that using a tray to transport breakable items at work was safer as he was able to grasp the tray better than individual items (P‐09). Another person decided to join patient forums and actively searched for more information about managing CIPN symptoms (P05). Patients' changing perceptions, growing knowledge and familiarity with CIPN symptoms also facilitated reporting behaviours and helped patients make decisions about their future treatment. However, some patients could only look back and wish that they had reported their symptoms sooner. Unfortunately, such realisation often took place during the final doses of chemotherapy treatment when the symptoms were severe and potentially irreversible.

#### 3.1.2 Clinician perspectives

##### Relative insignificance of CIPN

CIPN was viewed as non‐urgent when compared to other life‐threatening chemotherapy side effects. Providing CIPN information alongside other side effects of chemotherapy was challenging for clinicians. They focused on information about more acute side effects of chemotherapy treatment such as nausea and vomiting, hair loss, and risk of infection (FN; 14, 21 and 22 August). For example, a patient who reported CIPN but was also pyrexial (high temperature) during a consultation was investigated immediately for infection (FN, 23 September). Informing them about CIPN was delayed because infection was potentially fatal. Chemotherapy nurses, regardless of their seniority, reported that when they explained chemotherapy side effects, they went through the side‐effects list, from top to bottom, from pre‐printed drug‐specific patient information sheets and the information on the chemotherapy consent checklist (C‐01, C‐06, C‐10). This strategy ensured they covered everything. CIPN was far down the list of side effects in these documents; and information about CIPN and its management were limited (FN, 22 August). In contrast, senior doctors and experienced nurses who were aware of the side effects of neurotoxic drugs gave information about CIPN without prompts from drug information sheets. They were also observed to be more comfortable with assessing and asking CIPN‐relevant questions (FN; 19 September, 4 November).

##### Focus on acute CIPN symptoms

There was more emphasis on acute CIPN than long‐term CIPN symptoms. Clinicians identified numbness, tingling and cold‐induced neuropathy as the main symptoms of CIPN. However, the management advice given to patients was focused largely on managing cold‐induced neuropathic symptoms (C‐01, C‐06, C‐10, C‐11, C‐13; FN 19 September). Clinicians from the rehabilitation unit, who provide support for managing long‐term CIPN symptoms, identified CIPN symptoms serendipitously, that is, when patients were referred for other reasons such as post‐surgical rehabilitation (C‐07, C‐15). One nurse admitted, ‘I know that they do something to do with pain with peripheral neuropathy. Physiotherapy, I am not 100% sure exactly. I don't know what the service is but that would be something that I would refer …’ (C‐01). Most clinicians showed awareness and knowledge about the possible permanence of CIPN symptoms. Some reported that they do not give information about the duration of CIPN symptoms because they lacked knowledge and every patient is different.

##### Dependence on patient reporting

Clinicians mentioned that ‘underreporting or overreporting of CIPN symptoms are dependent on patients’ (C‐04) and the only way to monitor CIPN was for patients to inform their clinicians ‘because the symptoms cannot be determined through blood tests or routine clinical examinations’ (C‐07, C‐09). They also relied on the person who performed the assessment to communicate this further to the clinical team (C‐06, FN).

##### Difficulties and challenges providing support

When asked to describe their experiences and feelings about assessing and managing CIPN, clinicians predominantly used negative words and phrases such as ‘difficult’, ‘tricky’, ‘frustrating’, ‘gives us a lot of headaches’, ‘reliance on patients to report their symptoms’, ‘cannot be seen’, ‘a problem’ and ‘not easy to manage’. Some clinicians also perceived that their colleagues were worried about frightening patients regarding CIPN as they might refuse treatment (C‐08). Several clinicians said that whilst they felt they could not offer anything, they were disappointed they could not do any more to help manage CIPN symptoms and felt that patients were left to manage their symptoms themselves (C‐14, C‐06).

### 3.2 Theme 2: Assessment and management of CIPN is disconnected

#### 3.2.1 Responsibility and reactive management

Although clinicians accepted CIPN management was everyone's responsibility, the main onus was still on the oncologist (C‐07, C‐01, C‐05, C‐08). All clinicians were aware of a dose reduction approach to manage it; it was the only CIPN management strategy that was mentioned. This was also evident during observations; nurses and pharmacists assessed CIPN severity primarily to check if dose‐reduction by oncologists was needed (FN; 19 September, 7 October, 4 November). Junior chemotherapy nurses who identified CIPN symptoms reported these to senior nurses, who then referred patients to their oncologist for dose re‐assessment (C‐06). The acute oncology team dealt with calls from patients about CIPN symptoms by advising them to discuss it with their oncologist in their next pre‐chemotherapy cycle clinic appointment rather than informing clinicians directly (FN, September 19).

#### 3.2.2 Lack of referrals and missed opportunities

Within clinical teams, sharing experience and knowledge of CIPN was passed from the senior to junior clinicians during their day‐to‐day work. However, at the time of data collection, the rehabilitation team noted that they ‘haven't had as many CIPN referrals because there hasn't been a neuropathy teaching event more recently’ (C‐07). When asked what could support their practice, one of the clinicians remarked, ‘what we don't have, or I feel like we don't have, is a set (CIPN) protocol, or a set person, or a set team that I know I can contact if I'm worried about someone’ (C‐14).

However, during observations, and as mentioned by participants, there were several examples of good practice and existing strategies and opportunities for addressing CIPN in study site. These are listed on Table 5.

**TABLE 5:** Existing strategies and opportunities for addressing CIPN in study site

Stage	Existing strategies and opportunities for addressing CIPN
Pre‐chemotherapy	‐ Several timepoints in the process when written and verbal CIPN information was given e.g. pre‐consent visit, during consent visit, pre‐chemotherapy consultation with the chemotherapy nurse and in every pre‐chemotherapy cycle clinic appointment
Assessment and support during chemotherapy	‐ Ongoing support from clinical nurse specialists who patients can call ‐ Acute oncology services contact details were provided at the start of chemotherapy during pre‐chemotherapy consultation ‐ Review of CIPN symptoms before each cycle in outpatient clinics by oncologist or senior nurse or specialist pharmacist ‐ Physiotherapist and/or occupational therapists present in some clinics ‐ Chemotherapy nurses document CIPN symptom assessment on patients' electronic records ‐ Electronic referral to rehabilitation team ‐ Referral to neurology services ‐Clinician or patient self‐referral for complementary therapies
Discharge	‐ Referral to generic rehabilitation service (physiotherapy and occupational therapy) where the team conducts an overall assessment ‐ Review by clinical nurse specialists at end of treatment using holistic needs assessment ‐ Discharge letter to patient's general practitioner
Rehabilitation	‐ Rehabilitation service is available
Other	‐ Reminders in place to identify high‐risk patients notes on clinical summary sheets such as ‘grade 3 peripheral neuropathy, review’, ‘mobility getting worse since FOLFOX’, ‘review neuropathy and consider dose reduction’. ‐ Senior clinicians highlighted high CIPN risk patients during pre‐clinic meetings and providing guidance to the team about management plans

Theme 2 emerged from the clinician interviews and was also informed by observational data from consultations. From the observational fieldwork, there were four main teams who were involved in CIPN assessment and management from consent to end of chemotherapy:

- Outpatient clinic clinicians: oncologist, clinical nurse specialist, senior cancer nurses, highly specialised oncology pharmacists,
- Chemotherapy unit clinicians: senior and junior chemotherapy nurses,
- Rehabilitation team: physiotherapists and occupational therapists,
- Complementary therapists: accessed by patients outside of standard routine.

Clinicians were aware patients could report their symptoms to any of these groups, but less experienced clinicians lacked CIPN knowledge and awareness of available services. In some observations, CIPN was assessed by clinicians, but management advice was not forthcoming (FN; 19 September, 7 October, 4 November). There was limited understanding of what each of the four teams does for patients with CIPN (C‐07, C‐09, C‐14); this resulted in an uncoordinated management approach. It was also unclear who should take a lead in non‐pharmacological approaches to mitigate the impact of CIPN symptoms (C‐07, C‐15, FN 19 September).

### 3.3 Theme 3: Patients and clinicians expect openness in symptom reporting, information provision and CIPN management

When it came to CIPN symptom reporting, information provision and management of CIPN symptoms, clinicians and patients expected openness (P‐01, C‐01, C‐05). Patients expected their clinicians to tell them about what could really happen, the possible long‐term impact and management options (P‐01, P‐05, P‐12). But in some cases, patient participants were only able to remember some information about CIPN (P‐03, P‐05, P‐11). On the other hand, clinicians who saw patients in outpatient appointments before, during and after treatment highlighted that because CIPN ‘symptoms were not visual, the patient has to monitor their symptoms’ (C‐01). They also suggested that patients keep a diary of their side effects and record how neuropathy affects them daily or if symptoms were troublesome (C‐04). They expected patients to be open and not to hide the severity of their CIPN symptoms (C‐08).

Senior clinicians recognise the need to help patients appreciate that dose reduction due to peripheral neuropathy was about safety (C‐08, C‐09; FN 25 July, 19 September). They highlighted that reducing chemotherapy doses in this context would be a joint decision; by giving patients the information they needed, patients could be part of making an important treatment decision (C‐01, C‐08, C‐09).

I'd say it would be a joint decision. I have had patients who have not wanted to, you know, to stop or reduce the dose and that is quite difficult. But then it is about having an adult conversation with that person and I suppose it's about being honest. So, you know I would tend to say to people, ‘It's no good us carrying on if you start falling over and you cannot walk properly, we do not want to leave you like that at the end of this treatment.’ (C‐08)

However, it was perceived by patients that CIPN was ‘not high on the priority list’ (P‐11) during chemotherapy consultations and ‘one of the less important side‐effects’ (P‐09). But for those who understood CIPN, engaging in a treatment decision was simple.

For me because of my understanding of how I use my hands and how I work. Making that decision [dose reduction] was quite straightforward, like I was quite determined I was resolute, made sure obviously that I had the right information, and I asked all the right questions, which is why they reduced some of my chemo (P‐12).

## 4 DISCUSSION

Our findings show how individual and shared CIPN perspectives and experiences of patients and clinicians directly or indirectly affect the patients' overall experience. To our knowledge, our study is the first to explore shared CIPN experiences and perspectives of patients and clinicians.

The findings highlight the need to address the lack of patients' awareness of CIPN, as well as that of their families, friends and work colleagues. Clinicians mentioned they gave CIPN information to patients before chemotherapy started—this was also observed in consultations—but patients forgot CIPN information quite quickly.

The findings suggest factors which may explain why few patients remember CIPN. Firstly, patients' perception of CIPN was affected by how CIPN was presented and the level of priority assigned by clinicians. In contrast to other chemotherapy side effects, CIPN was low on the list of priorities when side effects were discussed. Consent checklists, drug information sheets and treatment diaries that were used in practice contain limited information about CIPN symptoms and management. Patients remembered acute CIPN symptoms and how to manage these because of the greater emphasis given to them by clinicians. Long‐term CIPN symptoms were rarely discussed in consultations and thus not recalled by patients when asked. These issues are problematic because of their effect on patients' perception of CIPN as insignificant during the early part of treatment or an issue that can be dealt with later; such perceptions influenced their reporting behaviours. Indeed, some patients in our study were unaware of the importance of early reporting of CIPN symptoms to their clinicians.

Assessment and management of CIPN is mainly reliant on patients telling clinicians openly about their subjective symptoms. Thus, clinicians should find ways to improve information provision and assist patients in forming their knowledge and perception of CIPN. Illness representations or perceptions enable patients to make sense of their symptoms and guide any coping actions concerning CIPN (Leventhal et al., 2016) such as symptom‐reporting behaviours, accessing available support from clinicians and engagement in making treatment decisions.

Secondly, new information such as acute, chronic, motor or sensory CIPN symptoms can be confusing; drug‐specific symptoms of CIPN and the wide‐ranging nature of symptoms may contribute to misperceptions. Earlier research reported CIPN was initially mistaken as a symptom of other medical conditions by patients (Bakitas, 2007). The obscurity of symptoms is compounded by the varying knowledge and confidence of clinicians when giving information. Patients' CIPN experiences shaped their perceptions and increased their understanding of CIPN over time. This confirms how it is crucial that CIPN information is given before commencement of chemotherapy and is continuously reinforced throughout treatment and beyond treatment completion (Tofthagen et al., 2013).

Whilst patients' understanding of CIPN was lacking at the start of treatment, their experience allowed them to grasp the uniqueness of the CIPN experience. The ambiguity of symptoms (Tanay et al., 2017) and the lack of available resources to describe CIPN may also explain why some patients in this study were unable to remember whether CIPN was discussed by their clinicians. Clinicians, patients and researchers should explore new approaches to helping patients retain CIPN information. For example, actual patient experiences and descriptions of CIPN might add value and clarity for future patient and clinician psychoeducational interventions.

The findings highlight the need for better connectedness of CIPN support and communication. Echoing findings from earlier studies (Al‐Atiyyat & Banifawaz, 2018; Binner et al., 2011; Smith et al., 2014; Taylor & Tanay, 2020), clinicians in our study emphasised the need for a clear treatment and action plan for CIPN. An organisational protocol for addressing issues was lacking. Clinicians missed opportunities to refer patients to CIPN services due to limited awareness of what was available. Instead of working in silos, clinicians should consider a multi‐disciplinary approach and collaboration to develop a cohesive, proactive and individualised CIPN patient care plan. Consistent with earlier studies (Al‐Atiyyat & Banifawaz, 2018; Binner et al., 2011; Smith et al., 2014), clinicians reported limited knowledge and understanding about CIPN, especially among junior clinicians. This led to difficulties and challenges providing support and over reliance on oncologists for managing CIPN symptoms. These findings suggest the need for strengthening knowledge about acute and long‐term CIPN, and drug and non‐drug management strategies other than dose reduction. Increasing awareness of available CIPN support both in hospital and in the community among healthcare professionals—particularly chemotherapy nurses who see patients at every treatment cycle—should be prioritised.

## 5 LIMITATIONS

Whilst our findings are based upon in‐depth explorations of the experience of participants in this study, they were conducted in a single centre involving a small number of participants. Nonetheless, it is assumed that findings will be transferable to similar contexts, such as individuals with similar clinical and demographic characteristics and clinical experience. Another limitation is the limited ethnic diversity of participants. Although we were able to interview some patients from minority ethnic or cultural backgrounds, we did not observe patient–clinician consultations involving patients or clinicians from minority ethnic backgrounds. Future research should be directed towards recruiting participants from more diverse ethnic and cultural backgrounds. The presence of the lead researcher during consultations may have reminded clinicians about CIPN and consequently increased attention and discussions about CIPN. This was evident in earlier stages of the observation period but eased when the clinical team became used to the researcher's presence. Further, participants' interview responses may have been refined to signify co‐operation with the researcher. This limitation may have been mitigated by a conversational interview style and a convenient interview time and place, allowing participants to convey their experience and significant issues more openly.

## 6 CONCLUSION

Our study led to greater understanding and comparison of patients' and clinicians' experiences of provision of care, information and support for CIPN. A strength of our findings rests on our use of multiple qualitative methods. Observations allowed us to see clinician practices and patient–clinician interactions regarding CIPN that we would not have understood at greater depth through interviews alone. Insights into patient and clinicians' CIPN experiences and factors that affect patient perception of CIPN are valuable when considering how to develop and evaluate novel interventions to improve patient experiences of CIPN. Our findings will inform the next phase, a theory‐informed intervention co‐design for CIPN.

## FUNDING INFORMATION

This paper presents independent research funded by the National Institute for Health Research (NIHR Doctoral Research Fellowship, Mary Anne Tanay, DRF‐2018‐11‐ST2‐017). The views expressed are those of the authors and not necessarily those of the NHS, the NIHR or the Department of Health and Social Care.

## CONFLICT OF INTEREST

The authors declare that there is no conflict of interest.

## CONSENT TO PARTICIPATE

All study participants gave consent and signed a consent form.

## Supporting information


**Table S1** COREQ Checklist

## Data Availability

The datasets during and/or analysed during the current study are available from the corresponding author on reasonable request. The authors have full control of all primary data which are available upon request.

## References

[ecc13517-bib-0001] Al‐Atiyyat, N. M. , & Banifawaz, A. Z. (2018). Oncology nurses' knowledge, practice, and confidence toward chemotherapy‐induced peripheral neuropathy in. Jordan Saudi Medical Journal, 39(11), 1158–1163. 10.15537/smj.2018.11.23303 30397717 PMC6274657

[ecc13517-bib-0002] Bakitas, M. A. (2007). Background noise: The experience of chemotherapy‐induced peripheral neuropathy. Nursing Research, 56(5), 323–331. 10.1097/01.Nnr.0000289503.22414.79 17846553

[ecc13517-bib-0003] Bate, P. , & Robert, G. (2007). Bringing user experience to healthcare improvement. The concepts, methods and practices of experience‐based co‐design (pp. 1–83). Radcliffe Publishing Ltd.

[ecc13517-bib-0004] Beijers, A. , Mols, F. , Dercksen, W. , Driessen, C. , & Vreugdenhil, G. (2014). Chemotherapy‐induced peripheral neuropathy and impact on quality of life 6 months after treatment with chemotherapy. J Community Support Oncol, 12(11), 401–406. 10.12788/jcso.0086 25856013

[ecc13517-bib-0005] Binner, M. , Ross, D. , & Browner, I. (2011). Chemotherapy‐induced peripheral neuropathy: Assessment of oncology nurses' knowledge and practice. Oncology Nursing Forum, 38(4), 448–454. 10.1188/11.Onf.448-454 21708535

[ecc13517-bib-0006] Braun, V. , Clarke, V. , Hayfield, N. , & Terry, G. (2019). Thematic analysis. In P. Liamputtong (Ed.), Handbook of research methods in health social sciences (pp. 843–860). Springer Singapore. 10.1007/978-981-10-5251-4_103

[ecc13517-bib-0007] Cresswell, J. (2014). Research design: Qualitative, quantitative and mixed methods approaches (4th ed., pp. 183–213). SAGE.

[ecc13517-bib-0008] Gordon, B. S. , Gbadamosi, B. , & Jaiyesimi, I. A. (2018). The relationship between chemotherapy‐induced neuropathy and quality of life in breast cancer survivors. Journal of Clinical Oncology, 36(15_suppl), e22111–e22111. 10.1200/JCO.2018.36.15_suppl.e22111

[ecc13517-bib-0009] Gordon‐Williams, R. , & Farquhar‐Smith, P. (2020). Recent advances in understanding chemotherapy‐induced peripheral neuropathy. F1000Res, 9, 1–13. 10.12688/f1000research.21625.1 PMC7076330 32201575

[ecc13517-bib-0010] Green, J. , & Thorogood, N. (2018). Qualitative methods for health research (4th ed., pp. 113–311). SAGE.

[ecc13517-bib-0011] Jordan, B. , Margulies, A. , Cardoso, F. , Cavaletti, G. , Haugnes, H. S. , Jahn, P. , Le Rhun, E. , Preusser, M. , Scotté, F. , Taphoorn, M. J. , & Jordan, K. (2020). Systemic anticancer therapy‐induced peripheral and central neurotoxicity: ESMO–EONS–EANO clinical practice guidelines for diagnosis, prevention, treatment and follow‐up. Annals of Oncology, 31(10), 1306–1319. 10.1016/j.annonc.2020.07.003 32739407

[ecc13517-bib-0012] Knoerl, R. , Lee, D. , Yang, J. , Bridges, C. , Kanzawa‐Lee, G. , Lita Smith, G. , & Smith, L. (2018). Examining the impact of a web‐based intervention to promote patient activation in chemotherapy‐induced peripheral neuropathy assessment and management. Journal of Cancer Education, 33(5), 1027–1035. 10.1007/s13187-017-1200-0 28265863

[ecc13517-bib-0013] Knoerl, R. , Weller, E. , Halpenny, B. , & Berry, D. (2019). Exploring the efficacy of an electronic symptom assessment and self‐care intervention to preserve physical function in individuals receiving neurotoxic chemotherapy. Biomed Central Cancer, 18(1203), 1–13. 10.1186/s12885-018-5093-z PMC6278100 30514351

[ecc13517-bib-0014] Leventhal, H. , Phillips, L. A. , & Burns, E. (2016). The common‐sense model of self‐regulation (CSM): A dynamic framework for understanding illness self‐management. Journal of Behavioral Medicine, 39(6), 935–946. 10.1007/s10865-016-9782-2 27515801

[ecc13517-bib-0015] Loprinzi, C. L. , Lacchetti, C. , Bleeker, J. , Cavaletti, G. , Chauhan, C. , Hertz, D. L. , Kelley, M. R. , Lavino, A. , Lustberg, M. B. , Paice, J. A. , Schneider, B. P. , Lavoie Smith, E. M. , Smith, M. L. , Smith, T. J. , Wagner‐Johnston, N. , & Hershman, D. L. (2020). Prevention and management of chemotherapy‐induced peripheral neuropathy in survivors of adult cancers: ASCO guideline update. Journal of Clinical Oncology, 38(28), 3325–3348. 10.1200/jco.20.01399 32663120

[ecc13517-bib-0016] Nowell, L. S. , Norris, J. M. , White, D. E. , & Moules, N. J. (2017). Thematic analysis: Striving to meet the trustworthiness criteria. International Journal of Qualitative Methods, 16(1), 1–13. 1609406917733847. 10.1177/1609406917733847

[ecc13517-bib-0017] Park, S. B. , Goldstein, D. , Krishnan, A. V. , Lin, C. S. , Friedlander, M. L. , Cassidy, J. , … Kiernan, M. C. (2013). Chemotherapy‐induced peripheral neurotoxicity: A critical analysis. CA: A Cancer Journal for Clinicians, 63(6), 419–437. 10.3322/caac.21204 24590861

[ecc13517-bib-0018] Smith, E. M. , Campbell, G. , Tofthagen, C. , Kottschade, L. , Collins, M. L. , Warton, C. , … Visovsky, C. (2014). Nursing knowledge, practice patterns, and learning preferences regarding chemotherapy‐induced peripheral neuropathy. Oncology Nursing Forum, 41(6), 669–679. 10.1188/14.Onf.669-679 25355022

[ecc13517-bib-0019] Staff, N. P. , Grisold, A. , Grisold, W. , & Windebank, A. J. (2017). Chemotherapy‐induced peripheral neuropathy: A current review. Annals of Neurology, 81(6), 772–781. 10.1002/ana.24951 28486769 PMC5656281

[ecc13517-bib-0020] Tanay, M. A. L. , Armes, J. , & Ream, E. (2017). The experience of chemotherapy‐induced peripheral neuropathy in adult cancer patients: A qualitative thematic synthesis. Eur J Cancer Care (Engl), 26(5), 1–13. 10.1111/ecc.12443 26786536

[ecc13517-bib-0021] Taylor, C. , & Tanay, M. A. (2020). Assessing and managing chemotherapy‐induced peripheral neuropathy. Cancer Nursing Practice, 20(3), 1–8. 10.7748/cnp.2020.e1717

[ecc13517-bib-0022] Tofthagen, C. (2010). Patient perceptions associated with chemotherapy‐induced peripheral neuropathy. Clinical Journal of Oncology Nursing, 14(3), E22–E28. 10.1188/10.Cjon.E22-e28 20529785

[ecc13517-bib-0023] Tofthagen, C. , Visovsky, C. , & Hopgood, R. (2013). Chemotherapy‐induced peripheral neuropathy: An algorithm to guide nursing management. Clinical Journal of Oncology Nursing, 17(2), 8. 10.1188/13.CJON PMC5469440 23538249

