# Supplementary material for: Clinician and patient experiences when providing and receiving information and support for managing chemotherapy‐induced peripheral neuropathy: A qualitative multiple methods study
Source: Eur J Cancer Care (Engl). 2021 Oct 12;31(1):e13517. doi: 10.1111/ecc.13517 (PMC9286565; doi:10.1111/ecc.13517)

Supplementary Information

European Journal of Cancer Care

Clinician and patient experiences when providing and receiving information and support for managing chemotherapy-induced peripheral neuropathy: a qualitative multiple methods study

Tanay MAL, Robert G, Raffety AM, Moss-Morris R and Armes J

Corresponding author:

MAL Tanay

King’s College London

Email: mary.tanay@kcl.ac.uk

Table SI.1 COREQ Checklist


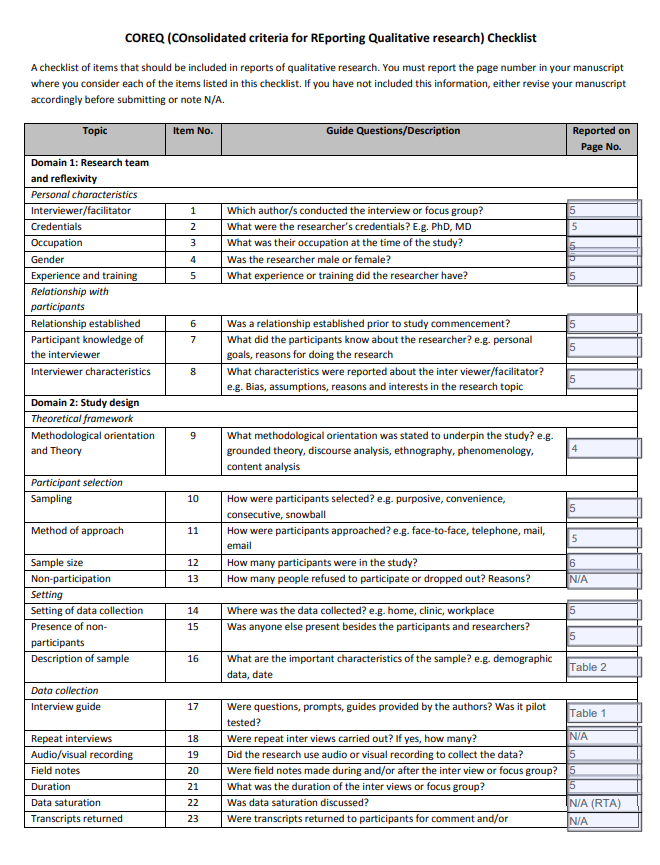


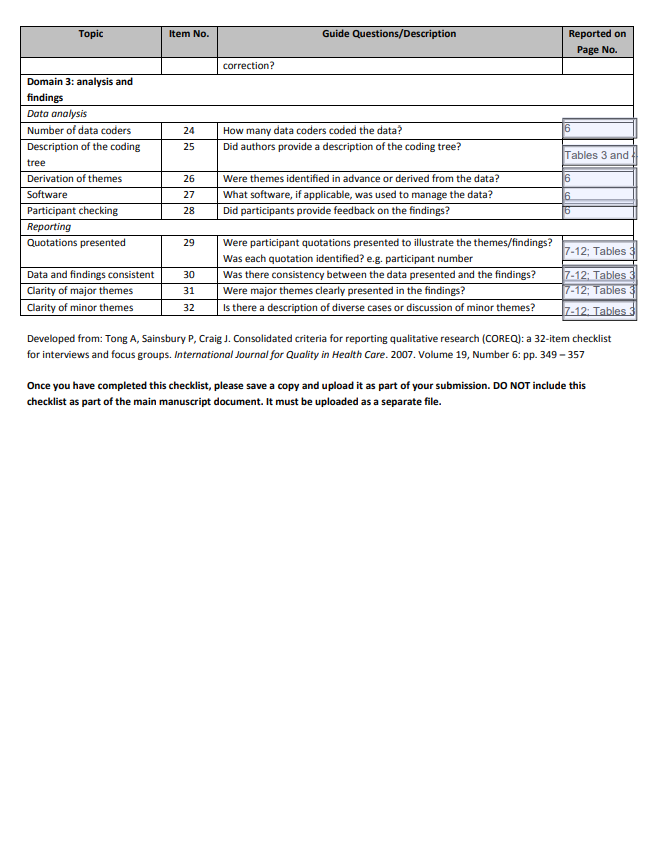

Supplement: Supplementary file 1 — Table S1 COREQ Checklist [file ECC-31-0-s001.docx]
